# 1-(4-Fluoro­phen­yl)thio­urea

**DOI:** 10.1107/S1600536810020246

**Published:** 2010-06-05

**Authors:** Aamer Saeed, Uzma Shaheen, Ulrich Flörke

**Affiliations:** a Department of Chemistry, Quaid-i-Azam University, Islamabad 45320, Pakistan; b Department Chemie, Fakultät für Naturwissenschaften, Universität Paderborn, Warburgerstr. 100, D-33098 Paderborn, Germany

## Abstract

In the title compound, C_7_H_7_FN_2_S, the aromatic ring plane and the thio­urea unit are twisted with a torsion angle C—C—N—C7 of 44.6 (2)°. In the crystal, N—H⋯S and N—H⋯F inter­molecular hydrogen bonds link the mol­ecules into infinite sheets that are stacked along the *c* axis.

## Related literature

For the biological activity of fluorinated thio­ureas, see: Sun *et al.* (2006 ▶); Saeed *et al.* (2009 ▶); Xu *et al.* (2003 ▶). For the use of fluorinated thio­ureas in organic synthesis, see: Nosova *et al.* (2006 ▶, 2007 ▶); Lipunova *et al.* (2008 ▶); Berkessel *et al.* (2006 ▶). *N*′-(2-fluoro­benzo­yl)thio­urea derivatives are suitable substrates for studying intra­molecular hydrogen bonds and Fermi resonance, see: Hritzová & Koščík (2008 ▶).
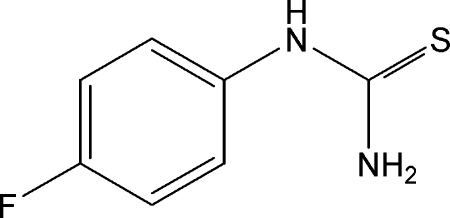


## Experimental

### 

#### Crystal data

C_7_H_7_FN_2_S
*M* *_r_* = 170.21
Monoclinic, 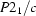

*a* = 9.1384 (8) Å
*b* = 8.4338 (7) Å
*c* = 10.5334 (9) Å
β = 109.796 (2)°
*V* = 763.85 (11) Å^3^
*Z* = 4
Mo *K*α radiation
μ = 0.37 mm^−1^
*T* = 120 K
0.43 × 0.39 × 0.29 mm

#### Data collection

Bruker SMART APEX diffractometer
Absorption correction: multi-scan (*SADABS*; Bruker, 2002 ▶) *T*_min_ = 0.857, *T*_max_ = 0.900
6816 measured reflections
1814 independent reflections
1645 reflections with *I* > 2σ(*I*)
*R*_int_ = 0.026

#### Refinement

*R*[*F* ^2^ > 2σ(*F* ^2^)] = 0.036
*wR*(*F* ^2^) = 0.097
*S* = 1.05
1814 reflections
101 parameters
H-atom parameters constrained
Δρ_max_ = 0.44 e Å^−3^
Δρ_min_ = −0.31 e Å^−3^

### 

Data collection: *SMART* (Bruker, 2002 ▶); cell refinement: *SAINT* (Bruker, 2002 ▶); data reduction: *SAINT*; program(s) used to solve structure: *SHELXS97* (Sheldrick, 2008 ▶); program(s) used to refine structure: *SHELXL97* (Sheldrick, 2008 ▶); molecular graphics: *SHELXTL* (Sheldrick, 2008 ▶); software used to prepare material for publication: *SHELXTL*.

## Supplementary Material

Crystal structure: contains datablocks global, I. DOI: 10.1107/S1600536810020246/pb2028sup1.cif

Structure factors: contains datablocks I. DOI: 10.1107/S1600536810020246/pb2028Isup2.hkl

Additional supplementary materials:  crystallographic information; 3D view; checkCIF report

## Figures and Tables

**Table 1:** Hydrogen-bond geometry (Å, °)

*D*—H⋯*A*	*D*—H	H⋯*A*	*D*⋯*A*	*D*—H⋯*A*
N1—H1*A*⋯S1^i^	0.88	2.43	3.2841 (12)	163
N2—H2*B*⋯F1^ii^	0.88	2.30	3.0989 (15)	152

Symmetry codes: (i) 
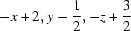
; (ii) 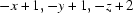.

**Fig. 1.**
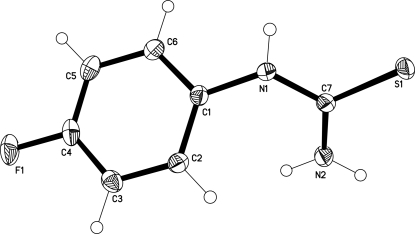
Molecular structure of title compound. Displacement ellipsoids are drawn at the 50% probability level.

**Fig. 2.**
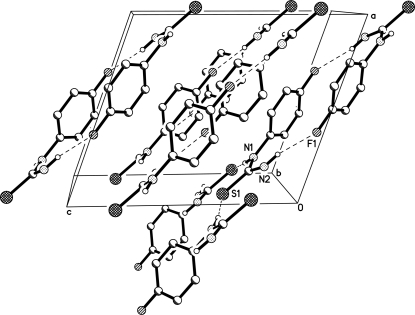
Crystal packing viewed along [001] with intermolecular hydrogen bonds indicated as dashed lines. H-atoms not involved in hydrogen bonding are omitted.

## supplementary crystallographic information

### Comment

Fluorinated thioureas are an imoprtant class of thioureas. Theser are versatile synthons for various fluorine-containing heterocycles: [1,3]-benzothiazin-4-ones (Nosova *et al.*,2006, 2007) 1-aryl-2-ethylthio-quinazolin-4-one, thiazolidine and 1*H*-1,2,4-triazoles (Lipunova *et al.*, 2008). Fluorinated thioureas exhibit a variety of biological activities: potent influenza virus neuraminidase inhibitors (Sun *et al.*, 2006), antimicrobial (Saeed *et al.*, 2009) and insecticidal activities (Xu *et al.*, 2003). Moreover, fluorinated bis-thiourea derivatives are also used as organocatalyst in Morita-Baylis-Hillman reaction (Berkessel *et al.*, 2006) and N-Substituted *N*'-(2-fluorobenzoyl)thiourea derivatives are suitable substrates for studying Intramolecular Hydrogen Bonds and Fermi Resonance (Hritzová & Koščík 2008). The aromatic ring plane and the thiourea moiety are twisted with a torsion angle C2–C1–N1–C7 of 44.6 (2)°. N(1)–H···S and N(2)–H···F intermolecular hydrogen bonds link molecules to endless 2D sheets that are stacked along the c axis.

### Experimental

4-Fluorobenzoylisothiocyante (1 mmol) in acetone was treated with ammonia (1 mmol) under a nitrogen atmosphere at reflux for 3 h. Upon cooling, the reaction mixture was poured into aq HCl and the precipitated product was rerystallized from in methanol to afforded the title compound (78 %) as colourless crystals: Anal. calcd. for C_7_H_7_N_2_O_2_FS: C, 49.40; H, 4.15; N, 16.46; S, 18.84%; found: C, 49.02; H, 4.17; N, 16.41; S, 18.91%.

### Refinement

Hydrogen atoms were clearly identified in difference Fourier syntheses, idealized and refined at calculated positions riding on the carbon atoms with isotropic displacement parameters U_iso_(H) = 1.2U(C/N_eq_).

### Crystal data

**Table:**

C_7_H_7_FN_2_S	*F*(000) = 352
*M**_r_* = 170.21	*D*_x_ = 1.480 Mg m^−^^3^
Monoclinic, *P*2_1_/*c*	Mo *K*α radiation, λ = 0.71073 Å
*a* = 9.1384 (8) Å	Cell parameters from 3596 reflections
*b* = 8.4338 (7) Å	θ = 3.2–28.3°
*c* = 10.5334 (9) Å	µ = 0.37 mm^−^^1^
β = 109.796 (2)°	*T* = 120 K
*V* = 763.85 (11) Å^3^	Block, colourless
*Z* = 4	0.43 × 0.39 × 0.29 mm

### Data collection

**Table:**

Bruker SMART APEX diffractometer	1814 independent reflections
Radiation source: sealed tube	1645 reflections with *I* > 2σ(*I*)
graphite	*R*_int_ = 0.026
φ and ω scans	θ_max_ = 27.9°, θ_min_ = 2.4°
Absorption correction: multi-scan (*SADABS*; Bruker, 2002)	*h* = −12→12
*T*_min_ = 0.857, *T*_max_ = 0.900	*k* = −10→11
6816 measured reflections	*l* = −13→12

### Refinement

**Table:**

Refinement on *F*^2^	Secondary atom site location: difference Fourier map
Least-squares matrix: full	Hydrogen site location: difference Fourier map
*R*[*F*^2^ > 2σ(*F*^2^)] = 0.036	H-atom parameters constrained
*wR*(*F*^2^) = 0.097	*w* = 1/[σ^2^(*F*_o_^2^) + (0.0546*P*)^2^ + 0.3192*P*] where *P* = (*F*_o_^2^ + 2*F*_c_^2^)/3
*S* = 1.05	(Δ/σ)_max_ = 0.001
1814 reflections	Δρ_max_ = 0.44 e Å^−^^3^
101 parameters	Δρ_min_ = −0.31 e Å^−^^3^
0 restraints	Extinction correction: *SHELXL97* (Sheldrick, 2008), Fc^*^=kFc[1+0.001xFc^2^λ^3^/sin(2θ)]^-1/4^
Primary atom site location: structure-invariant direct methods	Extinction coefficient: 0.009 (3)

### Special details

**Table:**

Geometry. All esds (except the esd in the dihedral angle between two l.s. planes) are estimated using the full covariance matrix. The cell esds are taken into account individually in the estimation of esds in distances, angles and torsion angles; correlations between esds in cell parameters are only used when they are defined by crystal symmetry. An approximate (isotropic) treatment of cell esds is used for estimating esds involving l.s. planes.
Refinement. Refinement of *F*^2^ against ALL reflections. The weighted *R*-factor wR and goodness of fit *S* are based on *F*^2^, conventional *R*-factors *R* are based on *F*, with *F* set to zero for negative *F*^2^. The threshold expression of *F*^2^ > σ(*F*^2^) is used only for calculating *R*-factors(gt) etc. and is not relevant to the choice of reflections for refinement. *R*-factors based on *F*^2^ are statistically about twice as large as those based on *F*, and *R*- factors based on ALL data will be even larger.

### Fractional atomic coordinates and isotropic or equivalent isotropic displacement parameters (Å^2^)

**Table:**

	*x*	*y*	*z*	*U*_iso_*/*U*_eq_	
S1	1.02935 (4)	0.53586 (4)	0.71645 (4)	0.02753 (15)	
F1	0.35073 (11)	0.23014 (12)	0.98827 (10)	0.0374 (3)	
N1	0.84161 (13)	0.39046 (13)	0.81980 (12)	0.0224 (3)	
H1A	0.8874	0.3066	0.8006	0.027*	
N2	0.85423 (14)	0.66261 (14)	0.84570 (13)	0.0257 (3)	
H2B	0.7864	0.6574	0.8882	0.031*	
H2C	0.8920	0.7550	0.8332	0.031*	
C1	0.71613 (14)	0.35888 (15)	0.86719 (13)	0.0195 (3)	
C2	0.57627 (15)	0.44244 (16)	0.81997 (14)	0.0214 (3)	
H2A	0.5656	0.5286	0.7594	0.026*	
C3	0.45251 (16)	0.39984 (17)	0.86130 (14)	0.0247 (3)	
H3A	0.3572	0.4568	0.8308	0.030*	
C4	0.47151 (16)	0.27309 (18)	0.94755 (14)	0.0254 (3)	
C5	0.60776 (17)	0.18705 (17)	0.99488 (14)	0.0261 (3)	
H5A	0.6167	0.0995	1.0538	0.031*	
C6	0.73102 (16)	0.23144 (17)	0.95434 (14)	0.0233 (3)	
H6A	0.8262	0.1745	0.9863	0.028*	
C7	0.89954 (15)	0.53115 (15)	0.80043 (14)	0.0206 (3)	

### Atomic displacement parameters (Å^2^)

**Table:**

	*U*^11^	*U*^22^	*U*^33^	*U*^12^	*U*^13^	*U*^23^
S1	0.0258 (2)	0.0168 (2)	0.0483 (3)	−0.00188 (12)	0.02351 (17)	−0.00191 (14)
F1	0.0391 (5)	0.0376 (5)	0.0475 (6)	−0.0133 (4)	0.0304 (4)	−0.0076 (4)
N1	0.0228 (5)	0.0154 (5)	0.0330 (6)	0.0021 (4)	0.0145 (5)	0.0018 (4)
N2	0.0267 (6)	0.0174 (6)	0.0386 (7)	−0.0017 (4)	0.0184 (5)	−0.0025 (5)
C1	0.0198 (6)	0.0188 (6)	0.0207 (6)	−0.0022 (5)	0.0080 (5)	−0.0014 (5)
C2	0.0233 (6)	0.0190 (6)	0.0222 (6)	0.0008 (5)	0.0080 (5)	0.0007 (5)
C3	0.0218 (6)	0.0250 (7)	0.0284 (7)	−0.0005 (5)	0.0101 (5)	−0.0055 (5)
C4	0.0272 (7)	0.0276 (7)	0.0262 (7)	−0.0106 (5)	0.0154 (5)	−0.0091 (5)
C5	0.0353 (7)	0.0230 (7)	0.0207 (6)	−0.0069 (6)	0.0103 (6)	0.0000 (5)
C6	0.0250 (6)	0.0201 (6)	0.0235 (6)	−0.0010 (5)	0.0066 (5)	0.0013 (5)
C7	0.0164 (6)	0.0189 (6)	0.0261 (7)	0.0003 (4)	0.0066 (5)	0.0014 (5)

### Geometric parameters (Å, °)

**Table:**

S1—C7	1.7035 (14)	C1—C2	1.3954 (18)
F1—C4	1.3616 (15)	C2—C3	1.3897 (19)
N1—C7	1.3427 (17)	C2—H2A	0.9500
N1—C1	1.4222 (15)	C3—C4	1.375 (2)
N1—H1A	0.8800	C3—H3A	0.9500
N2—C7	1.3274 (17)	C4—C5	1.380 (2)
N2—H2B	0.8800	C5—C6	1.3846 (19)
N2—H2C	0.8800	C5—H5A	0.9500
C1—C6	1.3899 (19)	C6—H6A	0.9500
			
C7—N1—C1	128.69 (11)	C2—C3—H3A	120.9
C7—N1—H1A	115.7	F1—C4—C3	118.71 (13)
C1—N1—H1A	115.7	F1—C4—C5	118.34 (13)
C7—N2—H2B	120.0	C3—C4—C5	122.95 (13)
C7—N2—H2C	120.0	C4—C5—C6	118.37 (13)
H2B—N2—H2C	120.0	C4—C5—H5A	120.8
C6—C1—C2	119.93 (12)	C6—C5—H5A	120.8
C6—C1—N1	117.80 (12)	C5—C6—C1	120.32 (13)
C2—C1—N1	122.03 (12)	C5—C6—H6A	119.8
C3—C2—C1	120.14 (13)	C1—C6—H6A	119.8
C3—C2—H2A	119.9	N2—C7—N1	119.76 (12)
C1—C2—H2A	119.9	N2—C7—S1	121.59 (10)
C4—C3—C2	118.28 (13)	N1—C7—S1	118.64 (10)
C4—C3—H3A	120.9		
			
C7—N1—C1—C6	−141.01 (15)	F1—C4—C5—C6	−179.50 (12)
C7—N1—C1—C2	44.6 (2)	C3—C4—C5—C6	0.5 (2)
C6—C1—C2—C3	0.9 (2)	C4—C5—C6—C1	−0.5 (2)
N1—C1—C2—C3	175.14 (12)	C2—C1—C6—C5	−0.1 (2)
C1—C2—C3—C4	−0.9 (2)	N1—C1—C6—C5	−174.66 (12)
C2—C3—C4—F1	−179.79 (12)	C1—N1—C7—N2	10.3 (2)
C2—C3—C4—C5	0.2 (2)	C1—N1—C7—S1	−169.25 (11)

### Hydrogen-bond geometry (Å, °)

**Table:**

*D*—H···*A*	*D*—H	H···*A*	*D*···*A*	*D*—H···*A*
N1—H1A···S1^i^	0.88	2.43	3.2841 (12)	163
N2—H2B···F1^ii^	0.88	2.30	3.0989 (15)	152

Symmetry codes: (i) −*x*+2, *y*−1/2, −*z*+3/2; (ii) −*x*+1, −*y*+1, −*z*+2.
